# Changes in pain catastrophizing predict later changes in fibromyalgia clinical and experimental pain report: cross-lagged panel analyses of dispositional and situational catastrophizing

**DOI:** 10.1186/ar4073

**Published:** 2012-10-25

**Authors:** Claudia M Campbell, Lea McCauley, Sara C Bounds, Vani A Mathur, Lora Conn, Mpepera Simango, Robert R Edwards, Kevin R Fontaine

**Affiliations:** 1 Department of Psychiatry & Behavioral Sciences, Johns Hopkins University School of Medicine; 5510 Nathan Shock Drive, Suite 100; Baltimore, MD 21224, USA; 2 Department of Anesthesiology, Perioperative and Pain Medicine, Harvard Medical School, Brigham & Women's Hospital; 850 Boylston Street, Suite 302; Chestnut Hill, MA 02467, USA; 3 Department of Health Behavior, University of Alabama School of Public Health; 1665 University Blvd; Birmingham, AL 35294, USA

## Abstract

**Introduction:**

Fibromyalgia (FM), characterized by wide-spread diffuse pain and sensory abnormalities, is associated with elevated indices of distress and pain-related catastrophizing compared to both pain-free samples and those with chronic pain conditions. Catastrophizing is a pervasive negative mental set, and is a strong predictor of negative pain-related outcomes such as clinical pain intensity, and physical disability. Situational catastrophizing, measured in the context of experimentally-induced pain, is strongly related to enhanced pain sensitivity, a core aspect of the pathophysiology of fibromyalgia. However, little is known regarding the temporal course of the association between catastrophizing and pain-related "outcomes". Most studies involve only static assessments of pain and catastrophizing at a single time point, which provides little insight into the direction of the observed associations. We sought to investigate the temporal relationships between catastrophizing and indices of both clinical pain (substudy 1) and experimentally-induced pain (substudy 2) in a larger randomized controlled longitudinal trial.

**Methods:**

Fifty-seven patients with FM completed catastrophizing, depression, and pain questionnaires as well as laboratory cold pressor pain testing at baseline, post-intervention and three month follow-up during a lifestyle physical activity study. Cross-lagged panel analyses were used to address these temporal relationships.

**Results:**

In substudy 1, analyses revealed that pre-to-post changes in dispositional catastrophizing ratings prospectively accounted for unique variance in subsequent post-to-follow-up changes in clinical pain ratings (p = 0.005), while pre-to-post changes in pain ratings did not account for unique variance in post-to-follow-up changes in catastrophizing ratings. An identical pattern was observed experimentally in substudy 2, with pre-to-post changes in situational catastrophizing ratings prospectively accounting for unique variance in subsequent post-to-follow-up changes in experimental pain ratings (p = 0.014), while pre-to-post changes in pain ratings did not account for unique variance in post-to-follow-up changes in catastrophizing ratings. Specifically, initial alterations in catastrophizing were associated with subsequent alterations in clinical and experimentally induced pain. Controlling for levels of depression did not affect the results.

**Conclusions:**

These findings provide empirical evidence that catastrophizing processes might precede and contribute to subsequent alterations in the pain experience for FM patients.

**Trial Registration:**

clinicaltrials.gov: NCT00383084.

## Introduction

Catastrophizing, a set of negative emotional and cognitive processes, is widely recognized as an important factor in amplifying chronic pain [1]. Dispositional catastrophizing, a trait-like measure, is associated with increased pain intensity ratings and greater numbers of tender points in patients with fibromyalgia (FM) [2]. Functional neuroimaging studies have also suggested that catastrophizing predicts individual differences in pain-related brain activations within multiple networks related to the processing of pain-related information [3]. Longitudinal research suggests that psychosocial factors such as catastrophizing are associated with increased risk of persistent pain [1], and related research findings demonstrate a variety of interventions that have been shown to reduce the severity of FM pain also decrease catastrophizing [4,5]. However, as the vast majority of relevant studies are cross-sectional, the temporal relationships between changes in pain and changes in catastrophizing have been difficult to fully elucidate. To date, it remains unclear whether reductions in catastrophizing influence subsequent pain responses, or whether treatment-associated decreases in pain drive the observed reductions in catastrophizing. In a multidisciplinary treatment study in patients with musculoskeletal pain, Burns and colleagues [6] found that early treatment-related changes in catastrophizing and pain helplessness (a component of catastrophizing) predicted late-treatment outcome changes in pain severity and functioning, but not vice-versa. To date however, no such analyses have been conducted in FM patients.

**Figure 1:**
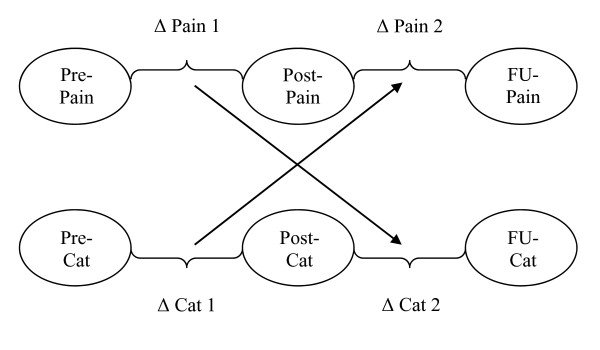
**Cross-lagged panel design**.

Situational catastrophizing measured during or immediately following laboratory pain procedures, is also strongly related to experimental pain ratings [7]. Our group has previously shown that early changes in catastrophizing may predict later changes in experimental pain ratings among healthy controls exposed to a tonically painful noxious stimulus [8]; however this relationship has not been examined prospectively in a clinical pain population. Herein, we evaluate the directional inter-relationships between longitudinal changes in pain and catastrophizing within a sample of FM patients. As in previous studies, we applied cross-lagged panel analyses [6,8-10], in this case, to data collected during a lifestyle physical activity intervention study in FM patients. Dispositional and situational catastrophizing, as well as clinical pain and experimental (cold pressor) pain responses, were measured at multiple points throughout the study, providing an opportunity to examine the temporal relationship of catastrophizing and pain over time. The cross-lagged panel analytic approach provides a technique to systematically characterize temporal associations between constructs of interest. We examined whether early changes in process (in this case catastrophizing) predict later changes in an outcome (pain), as well as the reverse-lagged associations (that is, whether early changes in pain predict subsequent changes in catastrophizing) and concurrent relationships [6]. Substudy 1 included dispositional catastrophizing and clinical pain, while substudy 2 included situational catastrophizing and experimental pain. See Figure 1 for a visual depiction of the study design.

## Methods

### Participants

FM patients were recruited for participation through the Johns Hopkins Arthritis Center, affiliated Johns Hopkins Rheumatology clinics, by newspaper advertisements, and clinical trial recruitment websites, including clinicaltrials.gov. Substudy 1 included a total sample size of 57 participants for analyses of clinical pain. Eight participants elected not to complete follow-up cold pressor testing, thus 49 are included in substudy 2, the experimental pain analyses. Mean age of the sample was 48.1 years (SD 11.5), and the group was predominantly female (95%) and non-Hispanic white (90%). The mean duration of FM was 7.4 years (SD 6.6). Those excluded from the experimental analyses were not significantly different from the larger group in any demographic category. Major inclusion criteria included age ≥ 18 years and who met American College of Rheumatology diagnostic criteria for FM [11]. Exclusion criteria included medical conditions that could preclude active participation (for example, cancer, coronary artery disease); those with the intention of altering their treatment, and those that were unwilling to make the required time commitment were excluded from the trial (see [12,13] for additional trial details). All study-related procedures were approved by the Johns Hopkins Hospital Institutional Review Board. Verbal and written informed consent was obtained upon arrival, after which participants underwent the assessment procedures described below.

### Study procedures

Participants were randomized to either a lifestyle physical activity (LPA) intervention group or a fibromyalgia education (FME) control group. LPA included six 60-minute group sessions over 12 weeks; sessions addressed FM-specific challenges to becoming more physically active, with a goal of accumulating 30 minutes of self-selected moderate-intensity physical activity five to seven days each week. The FME group met monthly for three months and included educational components and social support (see [12,13] for specifics of the interventions). All measures (described below) were collected at baseline, after the 12-week intervention and again at 3-month follow-up.

### Substudy 1

#### Clinical pain assessment

Pain was assessed using a 100-mm visual analogue scale (VAS) where participants rated their current level of pain, ranging from 0 (no pain) to 100 (worst pain imaginable).

#### Pain Catastrophizing

Participants completed the Pain Catastrophizing Scale (PCS) [14] at each of the three visits prior to undergoing any assessments. The measure consists of 13 items rated on a 5-point scale ranging from 0 (not at all) to 4 (all the time). Respondents indicate the degree to which they have specified thoughts and feelings when experiencing pain. The measure assesses three dimensions of catastrophizing: rumination, magnification, and helplessness. The PCS has been validated in both clinical and nonclinical samples [15].

### Substudy 2

#### Cold pressor pain assessment

Cold pressor pain was assessed by having participants immerse their nondominant hand up to the wrist in a 4°C water bath. Standardized instructions informed participants that they should maintain their hand in the water bath for 30 seconds; however if the pain became intolerable, participants were told that they could remove their hand at any time. Subjects were prompted to rate the intensity of the cold pressor pain using a 0 to 100 VAS. This procedure was conducted twice to ensure consistent readings. Pain ratings were averaged across the two trials for all subsequent analyses.

#### Situational Pain Catastrophizing

An adaptation of the Pain Catastrophizing Scale [14] used for a laboratory pain-testing environment was used to assess situation-specific catastrophizing as in prior studies from our group and others [7,16]. The Situation-Specific Pain Catastrophizing Scale (SPCS), administered immediately following the cold pressor pain testing, is a six-item questionnaire with responses ranging from 0 (not at all) to 4 (all the time). The scale has been described more fully by Edwards and colleagues [17] and its psychometric properties have been investigated [7]. In the current study, participants completed the catastrophizing questionnaire immediately following the second cold pressor procedure (described above). Participants were instructed to reference the cold pain they were experiencing in their hand while completing the questionnaire at each time point.

### Data reduction and analysis

Analysis of variance (ANOVA) was conducted to determine any overall effect of group (LPA vs. FME) on the variables of interest. Clinical pain ratings were used, and mean cold pressor pain ratings and summed dispositional and situational catastrophizing were calculated for each of the three time points. Zero-order correlations between both catastrophizing measures and pain measures at all three time pointes were computed. Three-factor repeated-measures ANOVA the factors being assessments made before treatment (Pre), immediately following 12-week treatment (Post), and at follow-up 3-months post-treatment (FU), were conducted to determine whether changes were observed from Pre assessments to Post and FU assessment epochs for dispositional and situational catastrophizing and clinical and cold pressor pain ratings. We computed standardized residualized change scores to index Pre-to-Post and Post-to-FU changes in each measure. We chose to use residualized change scores instead of simple change scores because of problems of dependence between change and Pre values encountered with use of the latter [18]. The use of residualized change scores has been recommended for cross-lagged panel analyses [9]. Next, we examined zero-order correlations between Pre-to-Post and Post-to-FU changes in catastrophizing and pain measures. Lastly, a series of hierarchical regression analyses were conducted to assess whether Pre-to-Post changes in each catastrophizing measure accounted for unique variance in the appropriate Post-to-FU change in pain ratings (clinical or cold pressor), or vice-versa, controlling for synchronous correlations and autocorrelations (that is, potential sources of extraneous variance). While these analyses were conducted on a convenient sample, our sample sizes were adequate to detect meaningful contributions of each variable at 80% power [9].

## Results

No group differences emerged by condition for any variable, with the exception of mean Post pain VAS score (LPA 45.9, SD 22.7; FME 62.1, SD 24.1; *P *= 0.01). As displayed in Table 1, Pre, Post and FU assessments of dispositional catastrophizing were inter-correlated (mean *r *0.79; *P *< 0.001), as were situational catastrophizing (mean *r *0.68; *P *< 0.001). Clinical pain was only significantly correlated at Pre and FU (*r *0.26; *P *= 0.05), while cold pressor pain ratings were correlated with each other at each time point (mean *r *0.46; *P *< 0.01). The average of the three concurrent correlations between PCS and SPCS (mean *r *0.26), between PCS and clinical pain (mean *r *0.26) and PCS and cold pressor pain (mean *r *0.14) was inconsistent. SPCS was correlated with clinical pain (mean *r *0.08) and cold pressor pain (mean *r *0.37), again with inconsistent degrees of significance. On average, clinical pain was marginally correlated with cold pressor pain (mean *r *0.14).

**Table 1:** Zero-order correlations between the PCS, SCS, clinical pain and experimental pain values at pre-, and post-treatment, and follow-up (FU) treatment time points

*N *= 57 (49 in Substudy 2)	1	2	3	4	5	6	7	8	9	10	11	12
1. Pre PCS (*n *= 57)	1.0											
2. Post PCS (*n *= 57)	0.80**	1.0										
3. FU PCS (*n *= 57)	0.78**	0.79**	1.0									
4. Pre SPCS (*n *= 49)	0.31*	0.34*	0.35*	1.0								
5. Post SPCS (*n *= 49)	0.20	0.26	0.31*	0.76**	1.0							
6. FU SPCS (*n *= 49)	0.08	0.16	0.28*	0.53**	0.74**	1.0						
7. Pre FM pain (*n *= 57)	0.20	0.30*	0.15	0.12	0.13	0.09	1.0					
8. Post FM pain (*n *= 57)	0.03	0.19	0.05	0.04	0.05	-0.06	0.23	1.0				
9. FU FM pain (*n *= 57)	0.38**	0.52**	0.49**	0.11	0.08	0.13	0.26*	0.20	1.0			
10. Pre CP pain (*n *= 49)	0.09	0.16	0.15	0.34*	0.39*	0.32*	-0.01	0.03	0.14	1.0		
11. Post CP pain (*n *= 49)	-0.08	-0.001	-0.06	0.17	0.38*	0.34*	0.38*	0.15	0.19	0.34*	1.0	
12. FU CP pain (*n *= 49)	0.27	0.35*	0.35*	0.37*	0.50**	0.48**	-0.04	0.08	0.24	0.54**	0.51**	1.0

*P < 0.05; **P < 0.01. FM, fibromyalgia; CP, cold pressor; PCS, Pain Catastrophizing Scale; SPCS, Situational Pain Catastrophizing Scale.

As shown in Table 2, repeated-measures ANOVA indicated that dispositional catastrophizing (*P *< .001) decreased significantly over time (from Pre-to-Post and Pre-to-FU). However, situational catastrophizing, clinical pain and cold pressor pain ratings did not substantially change over time.

**Table 2:** Pre- and post-treatment, and follow-up (FU) values for dispositional and situational catastrophizing and clinical and cold pressor pain ratings

Variable	Pre			Post			Follow-up			
	
	Mean	SD	*P *(Pre-Post)	Mean	SD	*P *(Post-FU)	Mean	SD	*P *(Pre-FU)	F value
PCS (*n *= 57)	21.37	12.39	< 0.001**	16.37	13.02	0.79	16.67	12.75	< 0.001**	13.23 (2,55)
SPCS (*n *= 49)	1.40	0.90	0.17	1.27	0.99	0.85	1.28	0.89	0.36	0.97 (2,47)
Clinical pain (*n *= 57)	55.48	24.86	0.72	54.02	24.56	0.68	52.34	23.95	0.43	0.30 (2,55)
Cold pressor pain (*n *= 49)	89.07	14.28	0.93	88.88	11.77	0.93	88.71	13.02	0.85	0.018 (2,47)

*P < 0.05; **P < 0.01. PCS, Pain Catastrophizing Scale; SPCS, Situational Pain Catastrophizing Scale.

### Cross-lagged hierarchical regression analyses

Correlations among standardized residual change scores are presented in Table 3. Two sets of hierarchical regressions were performed to determine whether (substudy 1) Pre-to-Post dispositional and change scores were significantly and uniquely associated with Post-to-FU changes in clinical pain, and (substudy 2) Pre-to-Post situational pain catastrophizing were significantly and uniquely associated with cold pressor pain ratings, or vice versa. In substudy 1, with Post-to-FU change in clinical pain as the criterion variable, change in clinical pain from Pre-to-Post and change in dispositional catastrophizing from Post-to-FU session accounted for 3% of the variance in the initial step of the regression. In the second step, Pre-to-Post change in catastrophizing accounted for a significant 14% of the variance. These data suggest that early changes in dispositional catastrophizing account for unique variance in later changes in clinical pain (Table 4).

**Table 3:** Zero-order correlation among the standardized residual change scores of PCS, SPCS, FM clinical pain, and cold pressor pain

Variable	1	2	3	4	5	6	7	8
1. Δ PCS 1	1.0							
2. Δ PCS 2	-0.28*	1.0						
3. Δ SPCS 1	0.07	0.15	1.0					
4. Δ SPCS 2	0.06	0.20	0.04	1.0				
5. Δ FM pain 1	0.22	-0.12	0.01	-0.15	1.0			
6. Δ FM pain 2	0.30*	0.16	-0.07	0.10	-0.07	1.0		
7. Δ CP pain 1	0.09	-0.32*	0.08	0.02	0.10	-0.23	1.0	
8. Δ CP pain 2	0.23	0.19	0.33*	0.16	0.04	0.28	-0.14	1.0

* *P *< 0.05. PCS, Pain Catastrophizing Scale; SPCS, Situational Pain Catastrophizing Scale; FM, fibromyalgia; CP, cold pressor; Δ PCS 1, Pre-to-Post PCS change; Δ PCS 2, Post-to-FU PCS change; Δ SPCS 1, Pre-to-Post SPCS change; Δ SPCS 2, Post-to-FU SPCS change; Δ FM pain 1, Pre-to-Post FM pain change; Δ FM pain 2, Post-to-FU FM pain change; Δ CP pain 1, Pre-to-Post CP pain change; Δ CP pain 2, Post-to-FU CP pain change.

**Table 4:** Summary of substudy 1, hierarchical regression analyses: cross-lagged regressions for dispositional catastrophizing predicting clinical pain and clinical pain predicting dispositional catastrophizing

Step	Variable	R	Adjusted R^2^	R^2 ^Change	F Change	Standardized β	*P*-value
**Δ Clinical Pain 2**							
1	Δ Pain 1	0.16	-0.01	0.03	0.75	-0.05	0.73
	Δ D cat 2					0.15	0.27
2	Δ D cat 1	0.41	0.18	0.14	8.72*	0.39	0.005
**Δ Trait Cat 2**							
1	Δ D cat 1	0.37	0.11	0.14	4.39	-0.36	0.01
	Δ Pain 2					0.26	0.05
2	Δ Pain 1	0.38	0.09	0.001	0.05	-0.03	0.83

*P < 0.05. Δ D cat 1, Pre-to-Post dispositional catastrophizing change; Δ D cat 2, Post-to-follow-up (FU) dispositional catastrophizing change; Δ Pain 1, Pre-to-Post clinical pain change; Δ Pain 2, Post-to-FU clinical pain change.

With Post-to-FU change in dispositional catastrophizing as the criterion variable, change in dispositional catastrophizing from Pre-to-Post and change in pain from Post-to-FU accounted for 14% of the variance in the initial step of the regression. In the second step, Pre-to-Post change in pain accounted for only 0.1% of the variance in Post-to-FU change in catastrophizing (Table 4).

In substudy 2, with Post-to-FU change in cold pressor pain as the criterion variable, change in cold pressor pain from Pre-to-Post and change in situational catastrophizing from Post-to-FU session accounted for 5% of the variance in the initial step of the regression. In the second step, Pre-to-Post change in situational catastrophizing accounted for a significant 11% of the variance. These data suggest that early changes in situational catastrophizing account for unique variance in later changes in cold pressor pain (Table 4).

With Post-to-FU change in situational catastrophizing as the criterion variable, change in situational catastrophizing from Pre-to-Post and change in cold pressor pain from Post-to-FU accounted for 3% of the variance in the initial step of the regression. In the second step, Pre-to-Post change in cold pressor pain accounted for only 0.2% of the variance in Post-to-FU change in situational catastrophizing (Table 5).

**Table 5:** Summary of substudy 2, hierarchical regression analyses: cross-lagged regressions for situational catastrophizing predicting cold pressor pain and cold pressor pain predicting situational catastrophizing

Step	Variable	R	Adjusted R^2^	R^2 ^Change	F Change	Standardized β	*P*-value
**Δ CP Pain 2**							
1	Δ CP pain 1	0.23	0.01	0.05	1.34	-0.15	0.31
	Δ S cat 2					0.18	0.20
2	Δ S cat 1	0.41	0.12	0.12	6.51*	0.35	0.014
**Δ State Cat 2**							
1	Δ S cat 1	0.19	-0.01	0.04	0.87	0.06	0.72
	Δ CP pain 2					0.16	0.29
2	Δ CP pain 1	0.19	-0.03	0.002	0.07	0.04	0.79

* *P *< 0.05. Δ S cat 1, Pre-to-Post state catastrophizing change; Δ S cat 2, Post-to-follow-up (FU) state catastrophizing change; Δ CP pain 1, Pre-to-Post cold pressor change; Δ CP pain 2, Post-to-FU cold pressor pain change.

Controlling for condition and depression, by entering it into the first block of each regression did not alter the pattern of results or significance level, thus condition was removed as a variable of interest.

## Discussion

This study investigated whether changes in dispositional and situational pain catastrophizing prospectively influence subsequent clinical and experimental pain, or vice versa. These findings add to a growing literature on prospective associations between catastrophizing and pain. Using a cross-lagged panel approach, the change in dispositional catastrophizing (about pain in day-to-day-life) from Pre-to-Post assessment was associated with (subsequent) changes in Post-to-FU clinical pain. The same relationship was observed for situational catastrophizing (about cold pressor experimental pain). These findings were observed even when controlling for Post-to-FU changes in catastrophizing and Pre-to-Post changes in pain (both clinical and experimental). In contrast, changes in clinical and experimental pain from Pre-to-Post were not associated with later changes in Post-to-FU catastrophizing. These results provide additional evidence that changes in catastrophizing might precede changes in pain response. Our group previously demonstrated this association in healthy participants undergoing laboratory capsaicin-induced pain [8]. This approach has also been employed by Burns and colleagues, [6,19] who similarly found prospective associations of changes in catastrophizing with changes in pain-related variables in the context of multidisciplinary pain treatment. Their results indicated that early treatment reductions in pain helplessness (a dimension of catastrophizing) predicted later treatment decreases in pain and interference, and early treatment reductions in catastrophizing and pain-related anxiety preceded later treatment improvements in pain severity, but not vice versa [19]. They also found that early-treatment changes in catastrophizing and pain helplessness predicted final treatment pain outcomes, even controlling for depression [6].

Conventionally measured catastrophizing is thought to reflect a dispositional trait that appears to be relatively stable over time in the absence of intervention [20-22]. Measurement of catastrophizing is typically assessed prior to laboratory pain induction procedures, when subjects complete one or more questionnaires that ask them to reflect and report on how much they generally catastrophize when in pain. In the current analyses, dispositional pain catastrophizing was reduced over time (to a similar degree in both treatment groups), and this reduction appears to have influenced a reduction in clinical pain reporting. Interestingly, recent reports suggest that dispositional assessment of catastrophizing may not be as relevant to experimentally induced pain [23]. Situational catastrophizing is assessed during or immediately following experimental noxious stimulation and refers the participant to the pain experienced during testing. A growing body of literature has noted the strong association between situational catastrophizing and pain ratings in healthy participants as well as populations with chronic pain [24-28], and several reports suggest that dispositional and situational measures are only moderately correlated [16,23,29]. The current findings suggest that alterations in Pre-to-Post situational catastrophizing, even in a chronic pain population, influence experimental pain reporting from Post-to-FU visits.

A growing collection of studies have shown that catastrophizing is linked to a number of pain-related symptoms in FM patients, including pain severity, disability and tender point counts [1]. Catastrophizing appears to be one of the key psychosocial factors in shaping pain and pain-related outcomes. For example, studies in patients with spinal pain have indicated that catastrophizing is the single most important pre-treatment risk factor that predicts poor outcomes of pain-relieving interventions [30,31]. Indeed, recent evidence suggests that catastrophizing, which is correlated with more general measures of distress and negative effect, may be the principal psychosocial driver of persistent pain symptoms. In a sample of over 200 women undergoing hysterectomy, pre-surgical anxiety level was initially a highly significant predictor of pain intensity at 48 hours after surgery, but after the inclusion of catastrophizing in the model, the influence of anxiety was no longer significant while catastrophizing remained in the multivariate model, fully mediating the effects of anxiety (*P *< 0.001) [32].

Several studies have found no association between dispositional and situational catastrophizing [7,16]. Unlike previous studies, our findings suggest that traditionally measured (dispositional) catastrophizing was associated with situational catastrophizing and both clinical and experimental pain at different time points. This finding is not surprising, given the relationship between catastrophizing and pain in populations with FM and those with other functional pain syndromes [1]. While prior psychometric studies have provided evidence for the reliability of catastrophizing measures, we were encouraged to observe fairly strong correlations over time for dispositional and situational measures, suggesting the relative stability of individual differences on these measures. Furthermore, it is interesting to speculate that a common influence of catastrophizing on both clinical and experimental pain responses might underlie the increasingly-documented association between individual differences in pain sensitivity and reported clinical pain. For example, the well-documented relationship between quantitative sensory testing (QST) responses and post-operative pain [33], or other indices of clinical pain severity [34] could be partially mediated by catastrophizing.

Recent studies have suggested that the pain-reducing effects of a variety of analgesic treatments, including non-psychological interventions, are due partially to their effects on cognitive-emotional processes such as catastrophizing. For example, exercise- and activity-based physical therapy interventions are effective in reducing chronic low back pain, and their analgesic effects are substantially mediated by the decreases they produce in catastrophizing [35,36]. The present results add to this body of findings, suggesting that initial alterations in catastrophizing result in changes in patient's pain experience.

In terms of mechanisms by which early changes in catastrophizing shape later changes in pain, some prior reports highlight compliance and willingness to engage in physical activity. Helplessness, a key component of catastrophizing, correlates with less effective compliance with treatment recommendations [37,38], and prospective studies in patients with low back pain have revealed that those with high levels of negative effect and catastrophizing are most likely to engage in extended periods of bed rest, least likely to exercise, and most likely to become physically de-conditioned over time [39,40]. It is possible, therefore, that in the context of an LPA treatment study such as this one, early reductions in catastrophizing are related to better compliance with physical activity-promoting interventions, or perhaps, simply the willingness to participate in a clinical trial involving physical activity, resulting in later reductions in clinical pain. Future studies may wish to examine this relationship more closely.

It is interesting to speculate that treatments that specifically reduce catastrophizing might augment interventions such as LPA. Several studies indicate that cognitive behavioral therapy (CBT) and related interventions might reduce catastrophizing, with positive effects on later pain and functional outcomes. For example, Riddle and colleagues studied total knee replacement patients, selectively recruiting a highly distressed, high-catastrophizing group [41]. Following surgery, patients in the pain-coping treatment condition, relative to usual care, reported greater reductions in catastrophizing and lower pain and disability at 2-month follow-up. It is possible, though not clear in this particular report, that lowering catastrophizing resulted in enhanced patient engagement in physical therapy/training (PT) and exercise, leading to lower levels of pain and improved function in the medium-term postoperative period. A 2010 meta-analysis found that psychological treatments for fibromyalgia, and CBT in particular, showed the greatest effects when compared to drug and other pain treatments [5]. In a recent randomized controlled CBT trial specifically focused on reducing catastrophizing, Alda and colleagues [42] found significant reductions in global pain catastrophizing, increases in pain acceptance, global function and quality of life compared with pharmacological treatment and usual care. These findings highlight the potential salutary effects of reducing catastrophizing in fibromyalgia patients.

Several limitations should be considered in the context of this work. First, this constitutes a relatively small sample of patients, given that all included here had to complete all time points. Second, these data are pooled from two treatment groups (lifestyle physical activity and educational control) from one larger randomized controlled trial. While no differences were observed between groups at the final treatment visit, we are unable to determine the influences of treatment over the course of the study. Thus, it is unclear how treatment interacted with (reduced) catastrophizing and pain between and within groups. Dispositional catastrophizing was reduced over time, while situational catastrophizing remained relatively constant. However, fluctuations in situational catastrophizing significantly predicted alterations in cold pressor pain responses over time, despite this relative stability. This may be explained by variation in individual catastrophizing responses driving the influence over pain reporting [9,43]. In addition, the current analyses are unable to characterize whether catastrophizing had any influence over global functioning or if reductions in catastrophizing may improve functioning through its effects on pain. Assessment of these factors would be a valuable addition to the literature, as global functioning is of extreme importance in this population [5].

## Conclusions

These data are novel as few longitudinal studies have examined changes in catastrophizing and pain in FM patients. In addition, this study extends our previous work [7,8] and the work of others [6,16,19] in several ways. We previously found that early changes in catastrophizing predicted later changes in pain in a healthy sample undergoing capsaicin testing [8]. The current findings suggest that in FM patients, change in clinical pain catastrophizing from Pre-to-Post treatment was associated with subsequent changes in Post-to-FU clinical pain. The same relationship was observed for experimental pain catastrophizing on cold pressor experimental pain, replicating our previous experimental pain findings in a chronic pain population. Changes in clinical and experimental pain from Pre-to-Post treatment were not associated with later changes in Post-to-FU catastrophizing. These results provide additional evidence that changes in catastrophizing might precede and stimulate changes in pain response.

## Abbreviations

ANOVA: analyses of variance; CBT: cognitive behavioral therapy; FM: fibromyalgia; FME: fibromyalgia education; LPA: lifestyle physical activity; PCS: Pain Catastrophizing Scale; SPCS: Situation-Specific Pain Catastrophizing Scale; VAS: visual analogue scale.

## Competing interests

The authors declare that they have no competing interests.

## Authors' contributions

LC, LM, SB, and MS contributed to the collection, inputting and scoring/interpretation of data. KF and RE designed the study and participated in the oversight of the study. VM and RE contributed to the analyses, interpretation and writing. CC wrote the initial draft of the manuscript and conducted the final analyses. All authors contributed to the finalization and critical assessment of the manuscript and have given final approval of this version.
